# Biomechanical evaluation of ortho-bridge system and proximal femoral nail antirotation in intertrochanteric fractures with lateral wall fracture based on finite element analysis

**DOI:** 10.3389/fbioe.2024.1368492

**Published:** 2024-06-21

**Authors:** Yuntao Long, Na Liu, Xiaomeng Huang, Weiming Liang, Jianke Liu, Zhaozhao Huang, Yanhui Zhang, Wen Wang

**Affiliations:** ^1^ The First Affiliated Hospital of Guangxi University of Science and Technology, Guangxi University of Science and Technology, Liuzhou, Guangxi Province, China; ^2^ Tianjin Walkman Biomaterial Co., Ltd., Newton Laboratory, Tianjin, China; ^3^ Shandong First Medical University and Shandong Academy Medical Sciences, Jinan, Shandong, China; ^4^ Department of Orthopaedics, The First Affiliated Hospital of Shandong First Medical University and Shandong Provincial Qianfoshan Hospital, Jinan, Shandong, China

**Keywords:** lateral wall fractures, intertrochanteric fractures, ortho-bridge system (OBS), proximal femoral nail antirotation (PFNA), finite-element analysis, bone biomechanics

## Abstract

**Background:**

The integrity of the lateral wall in femoral intertrochanteric fractures significantly impacts fracture stability and internal fixation. In this study, we compared the outcomes of treating intertrochanteric fractures with lateral wall involvement using the ortho-bridge system (OBS) combined with proximal femoral nail antirotation (PFNA) versus simple PFNA from a biomechanical perspective.

**Methods:**

Finite-element models of femoral intertrochanteric fractures with lateral wall involvement were subjected to fixation with OBS combined with PFNA and simple PFNA. Von Mises stress measurements and corresponding displacement assessments for each component of the model, including the proximal femur and lateral wall, were used to evaluate the biomechanical effects of OBS fixation on bone and intramedullary nail stability.

**Results:**

Using PFNA alone to fix intertrochanteric fractures with lateral wall involvement resulted in von Mises stress levels on the lateral wall exceeding safe stress tolerances for bone growth. OBS fixation significantly reduced stress on the lateral wall of the femur and minimized the stress on each part of the intramedullary nail, reducing the overall displacement.

**Conclusion:**

In cases of intertrochanteric fractures with lateral wall involvement, PFNA fixation alone may compromise the biomechanical integrity of the lateral femoral wall, increasing the risk of postoperative complications. The addition of OBS to PFNA significantly reduces stress on the lateral femoral wall. Consequently, OBS should be considered for lateral wall fixation when managing intertrochanteric fractures combined with lateral wall fractures.

## 1 Introduction

With the rapid increase in the global elderly population and life expectancy, the number of hip fractures in this population is also increasing (Lindskog and Baumgaertner, 2004; Chang et al., 2020). In elderly individuals, hip fractures encompass both femoral neck fractures and femoral intertrochanteric fractures, with intertrochanteric fractures representing approximately 40% of all hip fractures (Adeyemi and Delhougne, 2019). Surgical intervention is the first line of treatment and includes extramedullary fixation, intramedullary fixation, and joint replacement. Extramedullary dynamic hip screw (DHS) fixation is the preferred option for stable intertrochanteric fractures. However, owing to the severe complications associated with DHS, it is not the preferred choice for unstable intertrochanteric fractures (Ecker et al., 1975; Haidukewych, 2009). In such cases, intramedullary nailing is necessary. Studies have reported favorable outcomes for intramedullary nail fixation (Tawari et al., 2015). Nevertheless, complications may arise after intramedullary fixation, including intramedullary nail fractures, screw blade withdrawal, and non-union. These complications are often linked to suboptimal fracture reduction, screw blade positioning, and inadequate screw blade length. They are closely related to the stability and integrity of the lateral intertrochanteric wall (Bohl et al., 2014; Yu et al., 2016).

Professor Gotfried first proposed the importance of the lateral wall in the prognosis of femoral intertrochanteric fractures in 2004, defining the lateral femoral wall as the proximal extension of the femoral shaft or the lateral femoral cortex distal to the vastus ridge (Gotfried, 2004). The intact lateral wall provides biomechanical and lateral support for the proximal femur, thus improving stability, whereas lateral wall fractures can lead to uncontrollable excessive fracture collapse and varus (Gotfried, 2004; Gupta et al., 2010). In 2018, the Orthopaedic Trauma Association improved the classification system for femoral intertrochanteric fractures by adding lateral intertrochanteric wall fractures (Meinberg et al., 2018). The integrity of the lateral wall is considered the main prognostic factor for hip intertrochanteric fractures (Hsu et al., 2013; Hsu et al., 2015; Gao et al., 2018). At present, intramedullary nail fixation is the preferred choice for patients with primary or iatrogenic lateral wall fractures. Nevertheless, intramedullary nail fixation alone may not adequately address the complete reconstruction of the fractured lateral wall. Enhanced fixation through devices such as ring ligation, additional screws, or plates is needed to reconstruct the fractured lateral wall (Mohamed et al., 2020). However, the internal fixation devices for lateral wall fracture reconstruction have not reached a unified standard.

**FIGURE 1:**
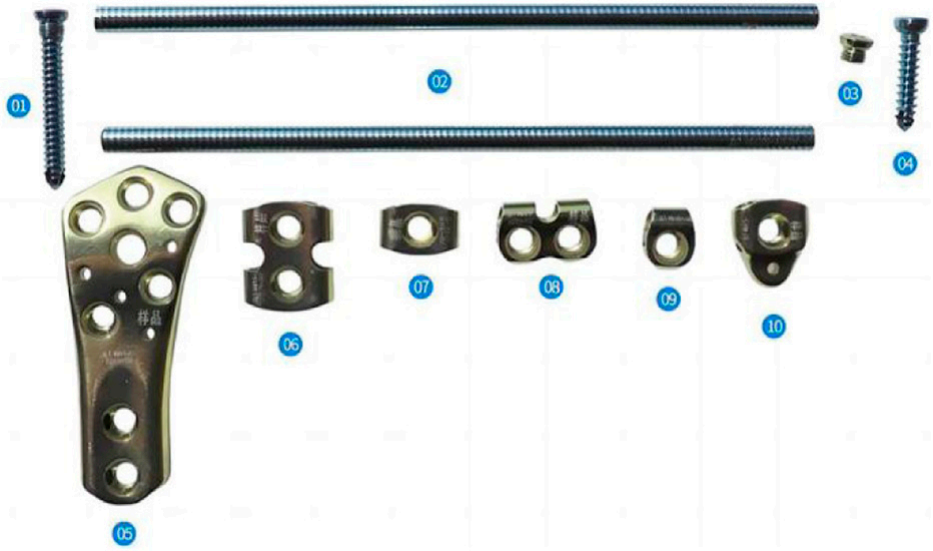
Basic unit components of the OBS. (01) Locking screws, (02) connecting rod, (03) locking nut, (04) ordinary screws, (05) distal shaped piece of the femur, (06) double-rod double-hole fixing block, (07) double-rod single-hole fixing block, (08) single-rod double-hole fixing block, (09) single-rod and single-hole fixing block, and (10) end block fixing block.

The ortho-bridge system (OBS) (Wang et al., 2014)—an original internal fixation system developed by China Tianjin Weiman Biomaterials Co., Ltd.—finds extensive clinical use in China for fracture fixation of the upper and lower extremities and pelvis fracture fixation. It consists of connecting rods, locking screws, and fixation blocks (Figure 1). Following the application of the bridge internal fixation system, combined with proximal femoral nail antirotation (PFNA) fixation for femoral intertrochanteric fractures accompanied by lateral wall fractures, we conducted a finite-element analysis to investigate the biomechanical changes of the lateral wall of the femur, proximal femur and intramedullary nail when OBS is used versus no OBS, as well as the mechanical advantages of OBS. These findings serve as a basis for future clinical applications.

## 2 Materials and methods

### 2.1 Finite-element model establishment

**FIGURE 2:**
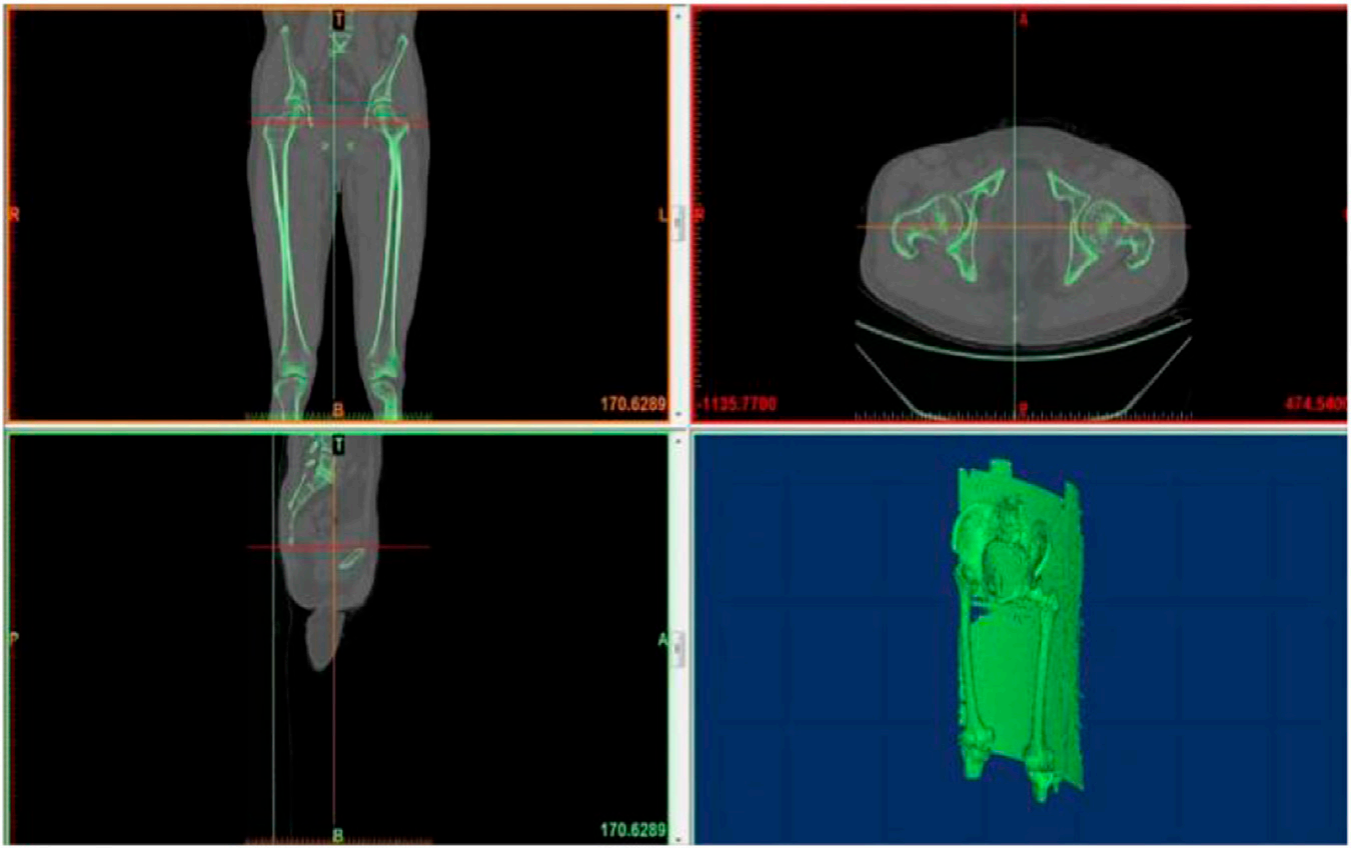
Femoral CT scan and 3D model image.

**FIGURE 3:**
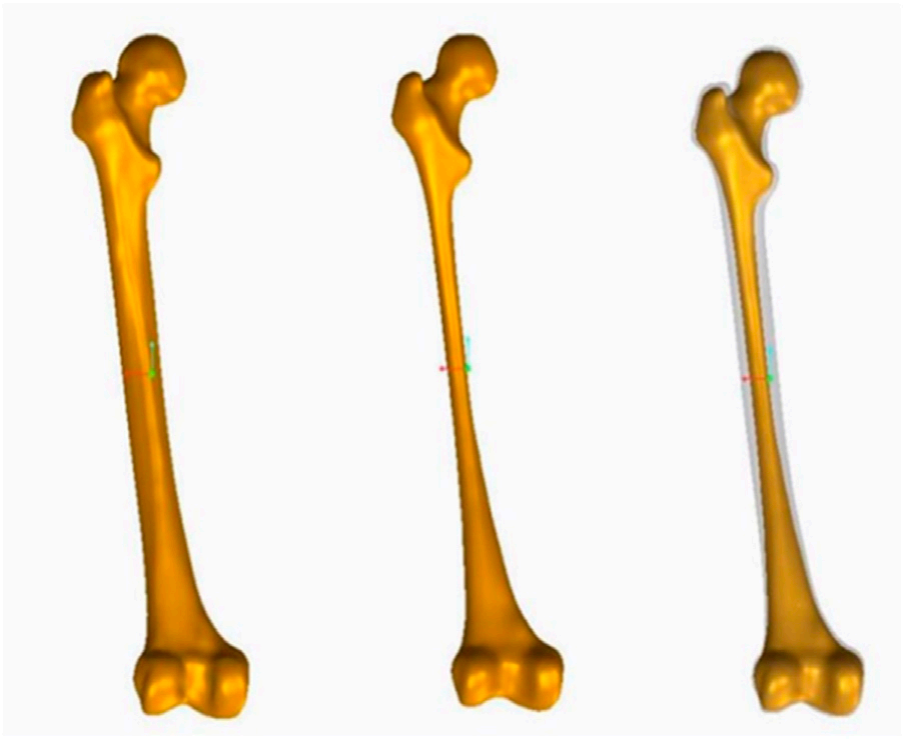
Geomagic precise fit surface model and 3D assembly diagram.

**FIGURE 4:**
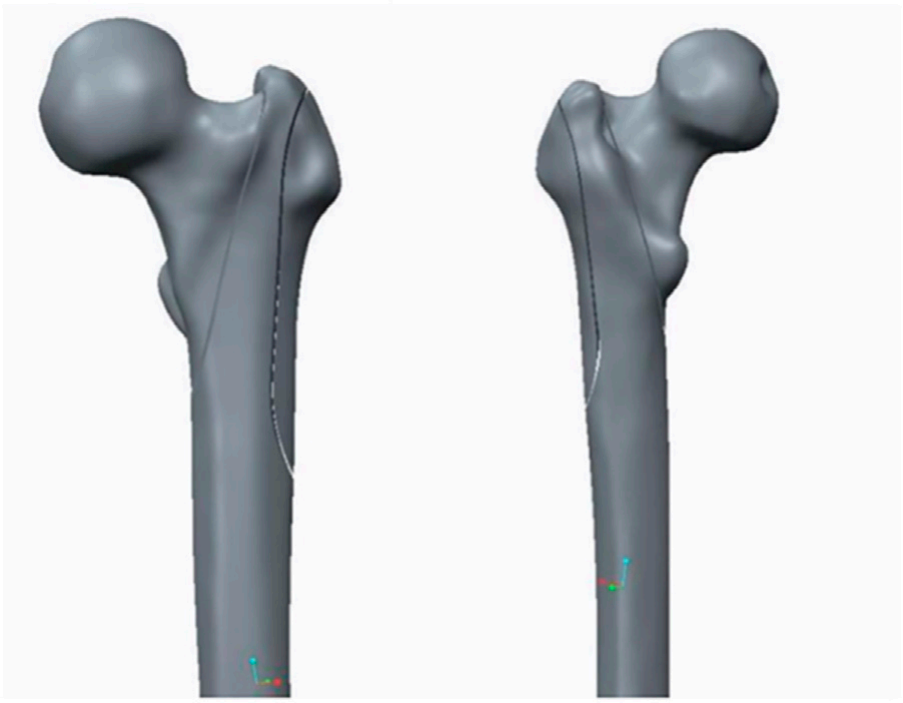
Anterior and posterior models of intertrochanteric and lateral wall fractures of the femur.

**FIGURE 5:**
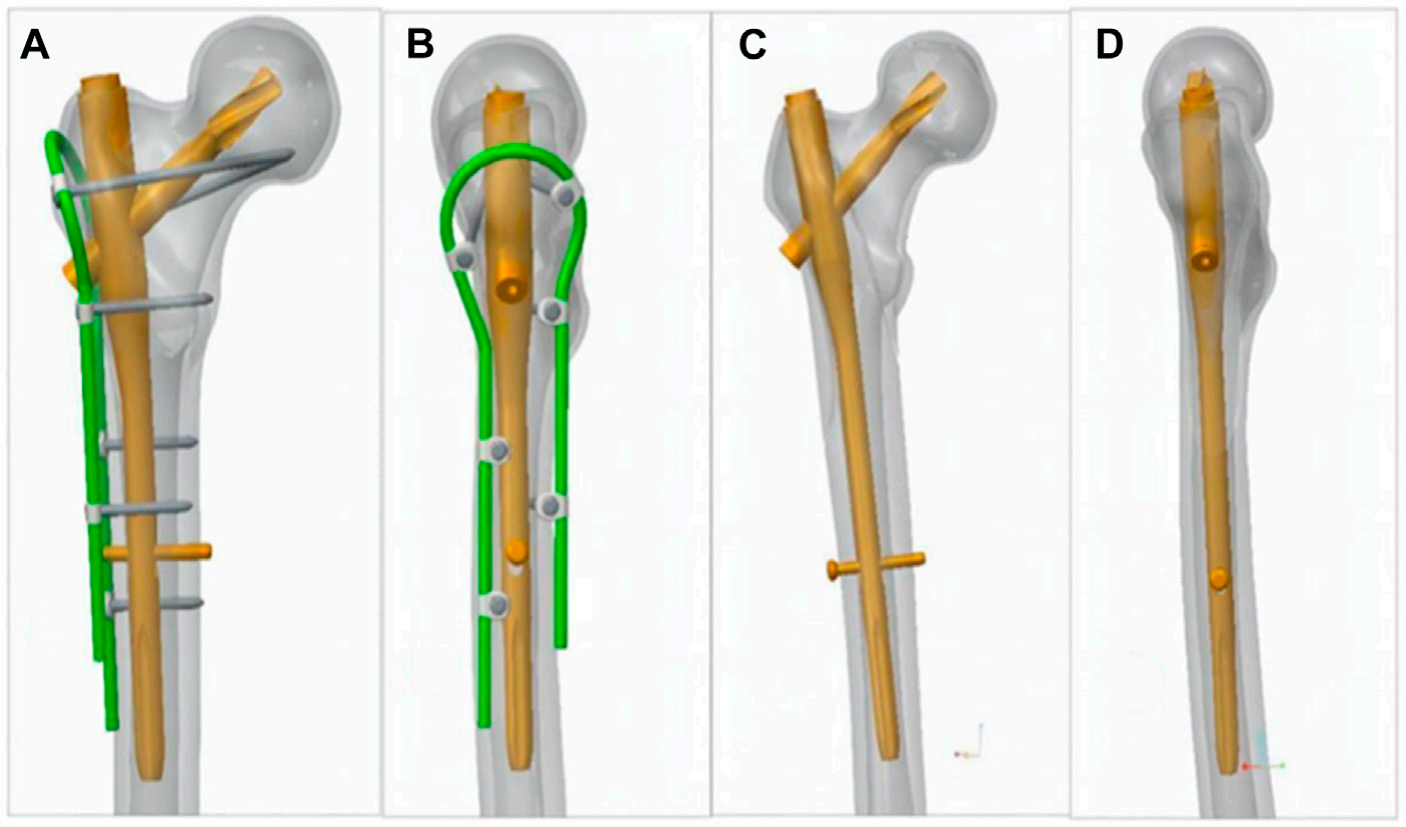
**(A, B)** OBS+PFNA fixed femoral intertrochanteric fracture combined with lateral wall fracture model and **(C, D)** PFNA only fixed fracture model.

The geometry of the femur model in this study was based on a computed tomography (CT) scan of a 68-year-old male volunteer who weighed 60 kg and had no femoral diseases. The femur’s CT scan data were imported into Mimics 19.0 software (Materialise, Belgium) to generate coronal and sagittal images of the proximal femur. Subsequently, a three-dimensional (3D) model of the proximal femur was reconstructed in IGS format (Figure 2). The cortical and cancellous bones of the femur were processed in the point, polygon, and shape stages using Geomagic Studio v2013 software (3D Systems, Rock Hill, SC, United States) to form the model of the cortical and cancellous bones of the femur. These bones were imported into the 3D model for assembly (Figure 3). The processed model, along with the required intertrochanteric and lateral wall fracture models, was imported into ANSYS Workbench19.0 software and constructed using its SLICE function (Figure 4). Creo 2.0 (Parametric Technology Corporation, United States) was used to load the PFNA and OBS into a 3D model in different manners (Figure 5).

In this study, all materials were assumed to have homogeneous isotropic elastic modulus distributions (Henschel et al., 2016). According to the CT images, the apparent densities of the cancellous and cortical bones of volunteers measured in Mimics were 397.75 and 1,518.10 kg/m^3^, respectively. According to the constitutive equation (Zheng et al., 2022) between the apparent density and elastic modulus, the elastic moduli of the cancellous and cortical bones were 1,389.700 and 10,551.347 MPa, respectively. Frictional contact described the contact interactions among bone fragments, implant components, and bones and implants. The friction coefficient between the cancellous and cortical bones was set to “bonded,” whereas that between the fracture surfaces was set to 0.46 (Li et al., 2019a). No friction existed between the intramedullary nail and the screw head. The contact areas between the implant and femur as well as the bridge internal fixation system were both set to “bonded.” All implant materials were made of titanium alloys. The characteristics of the femur and implant materials used in the model were summarized in Table 1 (Cun et al., 2020; Ding et al., 2021a; Ding et al., 2021b; Li et al., 2021).

**TABLE 1:** Material properties.

Material	Elastic modulus (MPa)	Poisson’s ratio
Cortical bone	10,551.347	0.3
Cancellous bone	1,389.700	0.3
Endophyte (titanium alloy)	110	0.33

### 2.2 Boundary and loading conditions

The distal femoral surface node was set to zero degrees of freedom, and a load was applied to the femoral head and greater trochanter to simulate a normal walking state. The load on the head of the femur was 10° to the vertical axis in the coronal plane and 9° (Li et al., 2019b) to the vertical axis in the sagittal plane, and a joint reaction force of 2,469.6 N (4.2 times body weight) was applied to the femoral head. A 1117.2 N abductor load (1.9 times the body weight) was applied at the greater trochanter (Fan et al., 2022).

ANSYS Workbench19.0 software was used for finite-element analysis, and this software was used to analyze the research results and to measure the von Mises stress and the corresponding displacement of the inner plant, proximal femur, and lateral wall of the model. The effects of OBS fixation on the biomechanics of the proximal femur and stability of the intramedullary nail were evaluated by comparing the measured data.

## 3 Results

### 3.1 Von Mises stress in proximal femur and lateral wall bone

**FIGURE 6:**
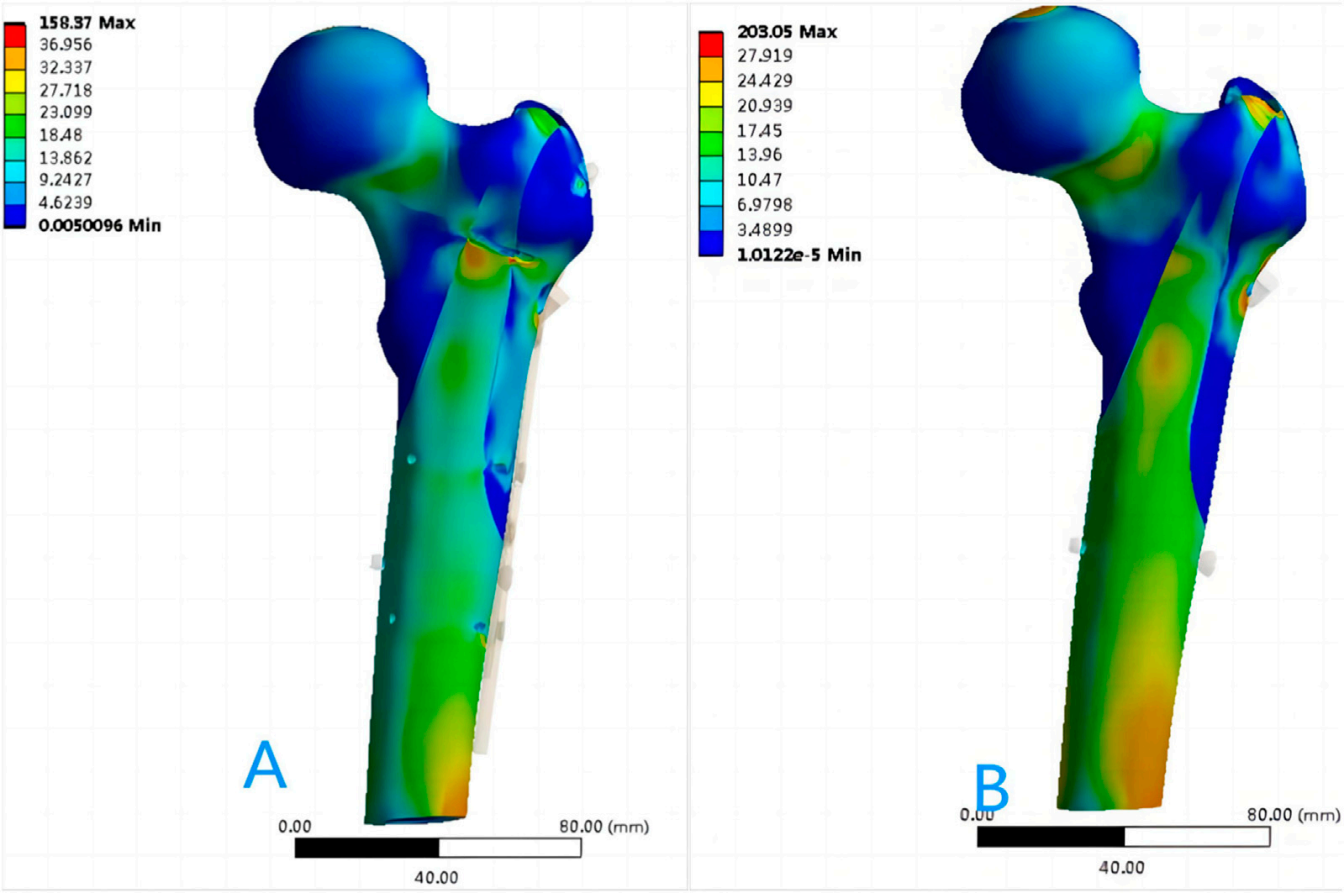
Distribution of von Mises stress in the proximal femur bone (MPa): **(A)** OBS+PFNA fixed model; **(B)** PFNA fixed model.

**FIGURE 7:**
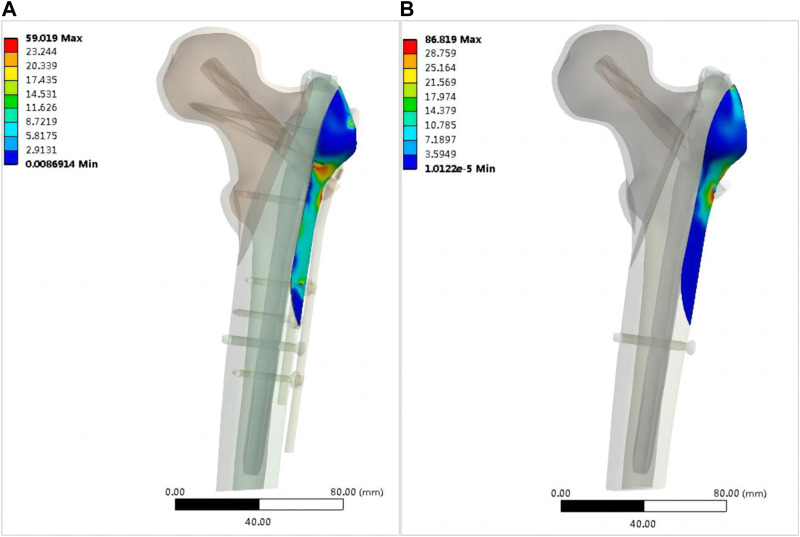
Stress distribution of the lateral wall skeleton (MPa): **(A)** OBS+PFNA fixed model; **(B)** PFNA fixed model.

Figure 6 shows the von Mises stress of the proximal femur fixed using OBS-assisted PFNA with a lateral wall fracture and simple PFNA fixation. The maximum stress of the proximal femoral bone of the double internal fixation model was 158.37 MPa, whereas that of the single internal fixation model was 203.05 MPa, which was 28.21% higher. The maximum stress of the double internal fixation model was concentrated in the bone area between the trochanters. The maximum stress in the single internal fixation model was concentrated at the opening of the screw blade. Figure 7 shows the stresses on the lateral walls of the two models. The maximum von Mises stress of the lateral wall bone in the double internal fixation model was 59.091 MPa, and the maximum stress of the lateral wall bone in the single internal fixation model was 86.819 MPa, corresponding to an increase of 46.92%.

### 3.2 Von Mises stress of components of intramedullary nail system and OBS system

**FIGURE 8:**
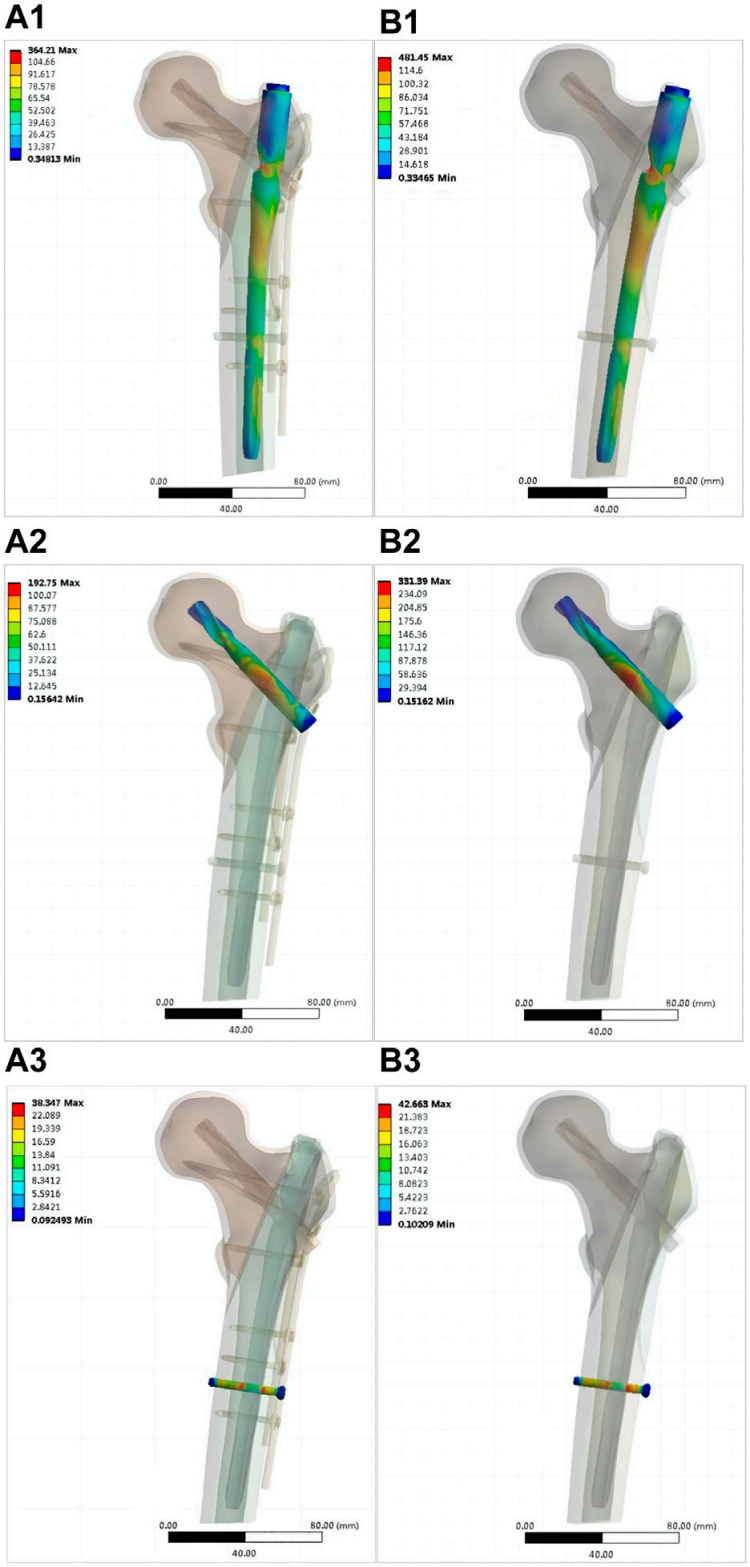
Distribution of von Mises stress in various components of intramedullary nails (MPa): OBS+PFNA fixation model [**(A1)** main nail, **(A2)** spiral blade, **(A3)** distal locking nail]; PFNA fixed model [**(B1)** main nail, **(B2)** spiral blade, **(B3)** distal locking nail).

**FIGURE 9:**
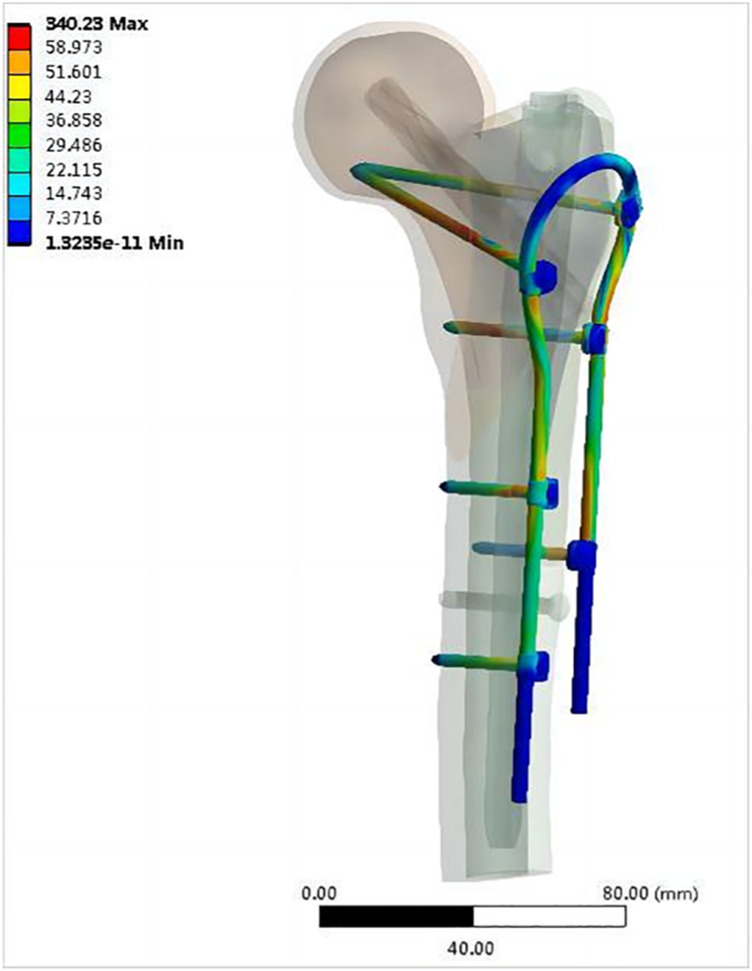
Von Mises stress distribution of the OBS (MPa).

Figure 8 shows the von Mises stress on each part of the intramedullary nail in both models. The maximum stress on the main nail of the double internal fixation model was 364.21 MPa, and that of the single internal fixation model was 481.45 MPa, corresponding to an increase of 32.19%. The maximum stress of the spiral blade of the double internal fixation model was 192.75 MPa, and that of the single internal fixation model was 331.39 MPa, corresponding to an increase of 71.93%. The maximum stress of the distal screw in the double internal fixation model was 38.347 MPa, and that in the single internal fixation model was 42.663 MPa; the increase was not significant. Figure 9 shows the von Mises stress of the OBS system. The maximum stress of the OBS system as a whole was 340.23 MPa. Stress was concentrated in the proximal femoral screw and connecting rod. Table 2 presents the von Mises maximum stress of the model as a whole, each part of the implant, and the bone.

**TABLE 2:** Von Mises maximum stresses of the overall model, various components of the internal plant, and bones.

Position	Maximum von Mises stress (MPa)	
	PFNA	OBS+PFNA
Overall model	481.45	364.21
Intramedullary nail	481.45	364.21
Helical blade	331.39	192.75
Distal locking nail	42.663	38.347
OBS	—	340.23
Proximal femur bone	203.05	158.37
Lateral wall fracture	86.819	59.091

### 3.3 Model displacement

**FIGURE 10:**
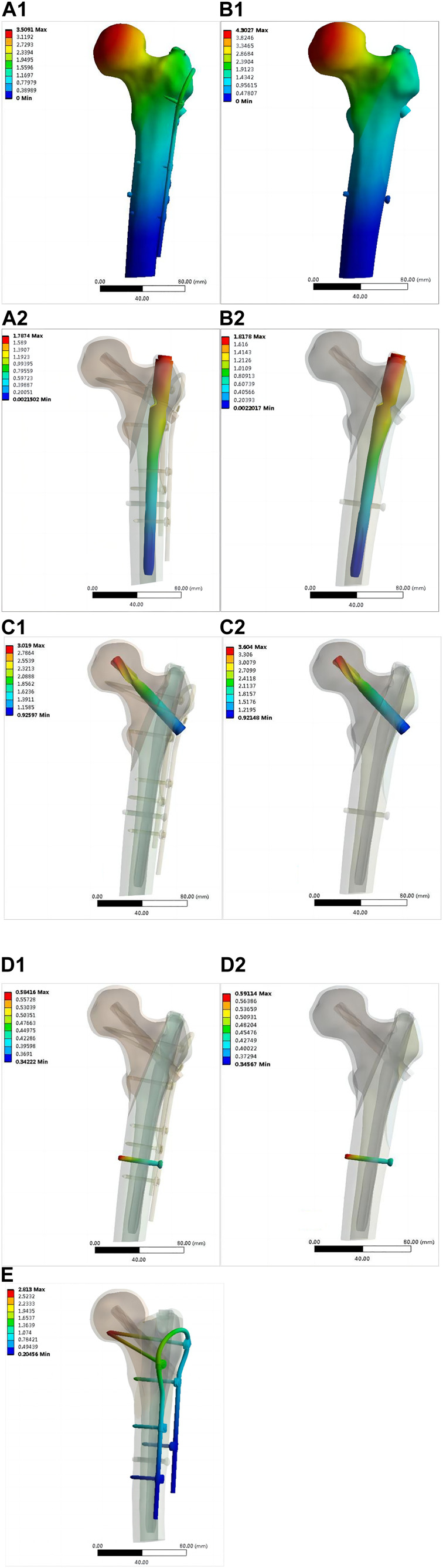
(Continued).

**FIGURE 11:**
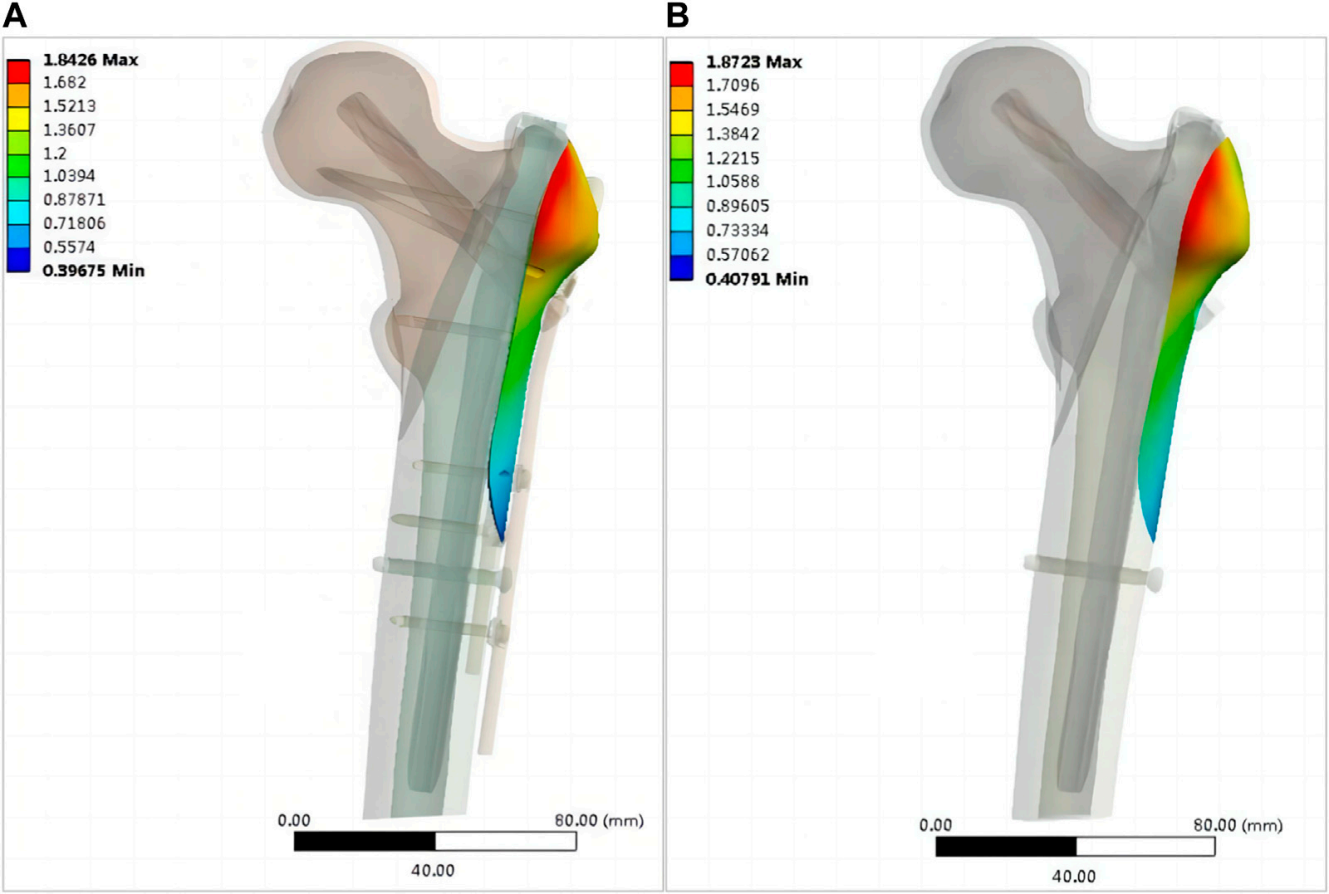
Distribution of bone displacement on the lateral wall of the model (mm): **(A)** OBS+PFNA fixed model; **(B)** PFNA fixed model.

Figure 10 shows the displacement of the entire fixation and each internal fixation in the two models. The maximum displacement of the double internal fixation model was 3.5091 mm, and that of the single internal fixation model was 4.3027 mm, corresponding to an increase of 22.6%. The maximum displacement of the main intramedullary nail of the double internal fixation model was 1.7874 mm and that of the single intramedullary nail was 1.8178 mm; the increase was not significant. The maximum displacements of the screw blade of the double internal fixation model, screw blade of the single internal fixation model, distal screw of the double internal fixation model, and distal screw of the single internal fixation model were 3.019, 3.604, 0.58416, and 0.59114 mm, respectively. No significant increase was observed, and the overall displacement of the OBS was 2.813 mm. Figure 11 shows the displacement of the lateral wall bone. The maximum displacement of the lateral wall bone of the double internal fixation model was 1.8426 mm, and that of the single internal fixation model was 1.8726 mm. No significant differences were observed.

## 4 Discussion

Treatment of femoral intertrochanteric fractures combined with lateral wall fractures is a difficult challenge for orthopaedic surgeons. Currently, there is no unified clinical standard on whether and how to fix the lateral wall. In this study, we tried to repair the lateral wall with OBS in order to increase the stability of intramedullary nail.

There are related clinical reports on the treatment of lateral wall fracture with steel wire or steel plate, Kulkarni SG et al. reported that fixing the lateral wall with steel wire can provide better stability and reduce complications such as fracture collapse (Kulkarni et al., 2017). Gupta RK et al. reported that the lateral wall fixation of 46 patients was performed using the trochanteric stabilising plate (TSP), and most of them recovered well. They believe that TSP ensures a better abductor function due to stability provided to the greater trochanter. However, there may be persistent pain in the hip region because of impingement of the proximal part of the trochanteric stabilising plate (Gupta et al., 2010). But there is no biomechanical report at present. Huang et al. reported the biomechanical analysis of the treatment of subtrochanteric fracture of femur with steel wire (Huang et al., 2021). Although the steel wire increased the local stress, it improved the stability of the medial femur. Encircling steel wire can also increase the stability of lateral wall, but whether to increase local stress during treatment needs biomechanical verification, and the biomechanical and clinical comparative study of internal fixators is needed to further improve the treatment of lateral wall reconstruction.

The instability of intertrochanteric fractures is often associated with the incomplete lateral wall of the femur. In such cases, intramedullary nail treatment becomes the preferred choice. Consequently, in this study, we employed PFNA fixation for the models. Significant variations in von Mises stresses on the lateral wall were observed between the two models. The maximum stress of the lateral wall of the double-fixation model was lower than 60 MPa, whereas that of the single-fixation model was 86.819 MPa. According to Wolff’s law and the study of Frost (Frost, 1994; Frost, 2004), bone tissue is absorbed when the strain is less than 50–100 microstrain and the stress is lower than 1–2 MPa. When the strain of the bone exceeds 1,000–1,500 microstrain and the stress is higher than approximately 20 MPa, the bone tissue grows. However, when the strain of the bone exceeds approximately 3,000 microstrain and the stress is higher than approximately 60 MPa, the bone tissue is damaged. Therefore, the use of OBS can effectively reduce the stress on the lateral wall and ensure that the bone tissue grows safely without stress shielding of the implant, whereas the von Mises stress of the lateral wall of the femur cannot grow well when the lateral wall of the femur is moved. Anatomically, the lateral wall is the lateral femoral cortex of the distal femoral crest, which provides lateral support (Varshney et al., 2022). Single-fixation refers to the loss of integrity of the lateral wall of the femur. When proximal femoral antirotation intramedullary nail-Asia (PFNA-II) is used, some scholars believe that the integrity of the lateral wall may not be important, because the metal lateral wall of the nail replaces the lateral cortex in osteoporosis (Boopalan et al., 2012; Abram et al., 2013). Studies by Shi et al. (2021) have shown that even when PFNA-II is used, the imaging and functional results of patients with lateral wall fractures are worse than those of individuals with intact lateral walls. Additionally, lateral wall fractures increase the risk of postoperative complications and reoperation (Palm et al., 2007). Gupta et al. considered that the strength of the lever arm and abductor muscle can be maintained when the lateral wall is reconstructed (Gupta et al., 2010); therefore, When conditions permit, the lateral wall of the femur should be fixed for fractures of the lateral intertrochanteric wall, whether it is a preoperative fracture or an intraoperative or postoperative intertrochanteric fracture, as reported by Gotfried (2004). In this study, the maximum von Mises stress of the proximal femur exceeded 60 MPa. However, Figure 6 shows that the distribution of the maximum stress was not near the fracture line, and the stress near the fracture line remains below 60 MPa, which does not hinder the normal healing of bones. Our findings suggest that the OBS device can effectively stabilize lateral wall fractures of the femur, thereby mitigating biomechanical deficiencies and lowering the risk of postoperative complications.

OBS affected the biomechanics of the intramedullary nail system and proximal femur bone in the model. In this study, the maximum von Mises stress was observed on the main nail of the single-fixation model. When the lateral wall of the femur was fixed with the OBS device, the maximum von Mises stress of the main intramedullary nail, the maximum von Mises stress of the screw blade, and the maximum stress of the proximal femur bone decreased significantly. Previous studies have shown that implants share part of the load borne by bone (Ridzwan et al., 2007). In the present study, we found that the implant can share not only the stress of the bone but also the load of each part of the intramedullary nail with internal fixation. As shown in Figures 7–9, no stress concentration was observed in the proximal femur. Part of the lateral wall of the femur belongs to the trabecular tension area of the proximal femur, and the force is tension (Stiehl et al., 2007; Nawathe et al., 2014). Internal fixation design must resist tension. Scholars (Ding et al., 2022) have developed the triangular support intramedullary nail to fix the intertrochanteric fracture of the femur, which proposes the triangular fixation theory of the proximal femur to better resist the tension in the femur. Internal plants placed in tension zones to resist the transverse tension of the femur, reduce the shear force, and reduce the stress on the intramedullary nail—particularly the shear force of the screw blade. This biomechanical feature can reduce the likelihood of postoperative internal fixation failure.

Currently the standard of peritrochanteric fracture treatment is stable fixation, which allows early full weightbearing mobilization of the patient (Macheras et al., 2012), However, most clinical patients may not be able to walk with full weight in the early postoperative period. The peak hip joint contact force during walking is 3.5–5 times of body weight, and studies conducted after hip prosthesis placement have shown that the peak hip joint contact force during the gait cycle is about 300% of body weight, but the maximum contact force reaches 409% of body weight in patients with gait disorders, and the hip joint peak force is higher when walking up and down stairs. In the case of unreasonable daily activities, such as accidental tripping, it can reach 900% (Bergmann et al., 2001). Therefore, patients must avoid unsafe activities in the early postoperative period. Based on previous studies, 4.2 times of body weight was applied to the femoral head (Fan et al., 2022). The peak joint reaction force during walking was selected for measurement to obtain the data at this limit state. In the follow-up study, it is possible to consider the actual weight of the patient and conduct a more detailed stratified load study, which may enhance the accuracy and comprehensiveness of the study.

In contrast to steel wires and plates, OBS can also be placed in a suitable position through shaping, but in OBS, the screw position is not completely fixed because the position of the connecting rod and fixing block is not fixed, and the screw is fixed according to the position of the fixing block, allowing us to place the nail flexible The position distribution of the screws is shown in the OBS model (Figure 5). Three screws are located in the front and back of the femur, and two screws are located at the proximal end, relative to the spatial distribution of the connecting rod and intramedullary nail; therefore, the lateral wall is fixed in multiple planes, and the length of screw placement exceeds that of the steel plate, even double-layer cortical fixation, and fixation is more reliable than single cortical fixation (Lenz et al., 2012; Lenz et al., 2016). We have combined OBS with PNFA in clinic, but there may be differences between the finite element analysis and the actual clinical use, and the analysis results need to be verified by clinical use in the future.

This study has several limitations. First, the femur and the implants exhibit anisotropic behavior. To reduce the complexity of the analysis, the materials were simplified as homogeneous, isotropic, and elastic materials. Second, our model exclusively conducts static analyses and does not encompass dynamic assessments. Consequently, future investigations should incorporate various forms of dynamic loading. Intertrochanteric fracture of femur mostly occurs in elderly women, so there may be some deviation in the research. In this study, we exclusively compared the biomechanical properties of OBS in conjunction with PFNA and simple PFNA fixation for intertrochanteric fractures with lateral wall involvement. To gain a more comprehensive understanding of lateral wall fixation options for the femur, including steel plate and steel wire fixation, further research is warranted. Additionally, we did not delve into the selection of subdivided internal fixation methods based on the classification of lateral femoral wall fractures in this study. Subsequent research efforts should explore the nuances of different types of lateral femoral wall fractures and the corresponding choice of internal fixation techniques.

## 5 Conclusion

We conducted a biomechanical comparison between OBS combined with PFNA and PFNA alone for treating femoral intertrochanteric and lateral wall fractures. OBS was found to effectively reduce stress on the lateral wall of the femur, promoting the safe growth of bone tissue without internal stress shielding. Moreover, it significantly reduced the maximum stress of the intramedullary nail and minimized the overall displacement of the model. The personalized design allows multiple-plane fixation of the femoral lateral wall. According to the findings of our study, we recommend the utilization of OBS fixing the lateral wall of the femur in cases of intertrochanteric fractures combined with lateral wall fractures. This approach promises to enhance the biomechanical stability of the fixation, potentially leading to improved clinical outcomes. Further clinical studies are encouraged to validate these biomechanical results and provide valuable insights into the practical implications of this fixation technique.

## Data Availability

The original contributions presented in the study are included in the article/supplementary material, further inquiries can be directed to the corresponding author.
